# A Multi-Class Automatic Sleep Staging Method Based on Photoplethysmography Signals

**DOI:** 10.3390/e23010116

**Published:** 2021-01-18

**Authors:** Xiangfa Zhao, Guobing Sun

**Affiliations:** College of Electronic Engineering, Heilongjiang University, Harbin 150080, China; 2181281@s.hlju.edu.cn

**Keywords:** PMSS, physiological signal, multimodal sleep staging, LightGBM

## Abstract

Automatic sleep staging with only one channel is a challenging problem in sleep-related research. In this paper, a simple and efficient method named PPG-based multi-class automatic sleep staging (PMSS) is proposed using only a photoplethysmography (PPG) signal. Single-channel PPG data were obtained from four categories of subjects in the CAP sleep database. After the preprocessing of PPG data, feature extraction was performed from the time domain, frequency domain, and nonlinear domain, and a total of 21 features were extracted. Finally, the Light Gradient Boosting Machine (LightGBM) classifier was used for multi-class sleep staging. The accuracy of the multi-class automatic sleep staging was over 70%, and the Cohen’s kappa statistic k was over 0.6. This also showed that the PMSS method can also be applied to stage the sleep state for patients with sleep disorders.

## 1. Introduction

Sleep plays a very important role in our daily life and is closely related to the operation of many physiological systems in the body. Poor sleep quality not only affects people’s daily life but also causes insomnia, narcolepsy, and other sleep disorders [1]. These acquired sleep disorders and congenital disorders are highly correlated with the duration of each sleep phase [2]. Not only that, sleep staging has been used to monitor the physiological status of some diseases in intensive care units, such as stroke, cardiovascular and cerebrovascular diseases, etc.

In the early study of sleep staging criteria, researchers divided sleep into non-rapid eye movement (NREM), wake, and rapid eye movement (REM) stages, and labelled rapid eye movement (REM) according to the state of the brain, blood pressure, heart rate, oxygen content in the blood, energy consumption, and other indicators of REM [3]. With the development of modern technology and further research on sleep, the non-rapid eye movement phase has been refined into sleep 1 (s1), sleep 2 (s2), sleep 3 (s3), and sleep 4 (s4) [4,5]. Among them, s1 and s2 are collectively referred to as light sleep, and s3 and s4 are collectively referred to as slow-wave sleep [6]. At present, the international standard staged sleep activity into five phases: REM, NREM I (N1), NREM II (N2), NREM III (N3), and wake (W) [7]. It is derived from researchers’ use of polysomnography (PSG) and the American Academy of Sleep Medicine (AASM) sleep scores and related event rulebook divisions [8,9]. The “gold standard” for assessing the sleep stage is a sleep staging method based on PSG technology consisting of multiple digital signals, including electroencephalogram (EEG), electrocardiogram (ECG), leg and chin electromyography (EMG), electrooculogram (EOG), respiration, oxygen saturation, and airflow. PSG technology is usually performed by multiple certified researchers by analyzing PSG signals for sleep staging at 30 s intervals [10].

Traditionally, sleep physiologists perform sleep staging by visually examining PSG signals. This method not only consumes expensive human resources but also relies on the professional level and experience of the evaluator [11]. In recent years, automatic sleep staging technology has liberated the limitations of manpower and improved the efficiency of sleep staging, which has become the main direction of people’s research at this stage and has achieved good results. In the early stage of the development of automatic sleep staging technology, researchers mainly used a combination of physiological signals to perform sleep staging, and the accuracy rate of 5-class sleep staging could reach more than 92% [12,13]. To reduce the impact of data collection on the subjects, the researchers turned to single-channel EEG signals, single-channel EOG signals, and single-channel ECG signals. Hassan and Bhuiyan used single-channel EEG signals to perform 5-class sleep staging with an accuracy of 91% [14]. Rahman et al. used single-channel EOG signals with over 90% accuracy for 5-class sleep [15]. Yücelbaş et al., Yoon et al., and Xiao et al. used ECG signals for 3-class sleep staging with an accuracy of more than 87% [7,16,17]. However, the above methods will affect the sleep state of subjects during the physiological signal collection process, and some methods must be performed in professional environments such as hospitals. To this end, Fonseca et al. and Beattie et al. tried to use the PPG signal extracted by the optical sensor for sleep staging and demonstrated the feasibility of the approach [18,19]. High-precision sleep staging and home sleep monitoring methods that reduce the impact on subjects become the next major goals for sleep staging researchers.

Here, the PMSS (PPG-based multi-class automatic sleep staging) method was proposed on a single-channel optical PPG signal for sleep staging. With less impact on the subjects during data collection, mobile monitoring and home monitoring can be easily achieved. In the CAP sleep database [20,21], PSG signals from more than 27,000 periods of 27 subjects were used to extract PPG signals as data, and the process of collecting physiological signal data would not affect the subjects’ natural sleep and will not cause psychological distress to the subjects. After preprocessing the data, feature extraction was performed from the frequency domain, time domain, and nonlinear domain. Finally, the Light Gradient Boosting Machine (LightGBM) classification model was used to perform sleep staging according to multiple classification principles. The classification results are described based on various indicators such as confusion matrix, accuracy rate, recall rate, F1 value, and Cohen’s kappa statistic. The method in this paper is suitable for family monitoring of different subjects, and the obtained results are equivalent to the results of PSG signal sleep staging.

## 2. Materials and Methods

PPG, based on a reflection-type detector based on an LED light source, measures the attenuation of reflected light, some of which is absorbed by human blood vessels and tissues. The pulse state of the blood vessel is then recorded, and the pulse wave is plotted. PPG signal can extract physiological signals such as heart rate, SpO2, and heart rate variability. This PPG-based test is usually applied to the fingertips, so it is safe, painless, and contains all the information needed for sleep staging and long-term monitoring. It is the first choice for portable sleep staging.

The PPG data collected in the CAP sleep database was abstracted to verify the PMSS method. The CAP sleep database is a collection of 108 polysomnography records registered by the Ospidere Marjorie Sleep Disorders Center in Palma, Italy. Using the Rechtschaffen and Kales (R&K) guidelines [8] and the AASM guidelines [9], several experts annotated all PSG records for a sleep phase every 30s and assigned the sleep phase to each data epoch. Due to the lack of PPG signal data in the PSG signal of 108 subjects, the sleep data of 27 subjects without PPG signal loss were used in this experiment, including 4 healthy subjects, 8 patients with REM sleep behavior disorder, 10 patients with nocturnal frontal lobe epilepsy, and 5 patients with insomnia. The subject information was extracted as shown in Table 1.

**Figure 1:**
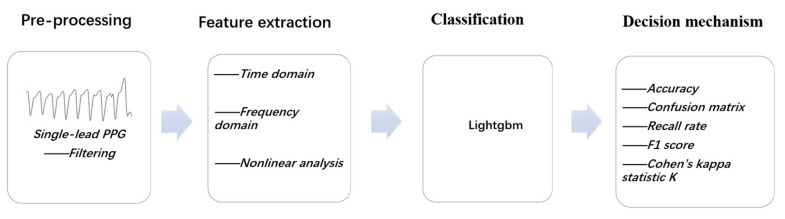
Flow diagram of automatic sleep staging mechanism.

In this study, small changes in PPG signals at different sleep stages were used to conduct multi-class sleep staging. Since there was be a lot of interference in the collection process of physiological signals, this study first preprocessed the PPG signals. Then, the features extracted from time-domain features, nonlinear-domain features, and heart rate variability signals were prepared for the preprocessed signals. Then, the frequency-domain features of heart rate variability were extracted. Finally, the extracted features were put into the machine learning model for sleep staging. The basic flow is shown in Figure 1, and the details of each step are described below.

### 2.1. Preprocessing of Raw Data

**Figure 2:**
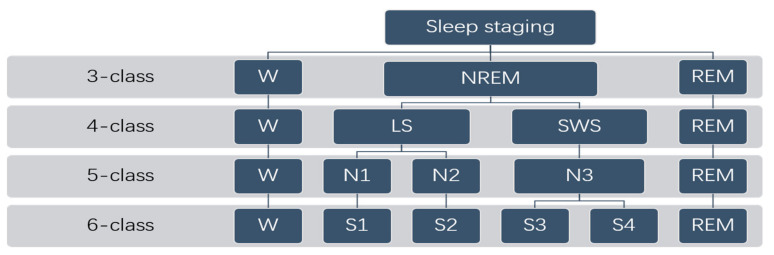
Multimodal sleep staging.

For noise, such as baseline drift and power frequency interference of PPG signal, the wavelet transform method was adopted in this experiment, and the BiOR3.5 wavelet was selected for filtering. At the same time, the 6 classification tags in the original data were converted into a multimodal tag, as shown in Figure 2. In addition, this study still used the division of sleep stage every 30 s in the original data and manually deleted some data without sleep state annotations, and finally obtained 27,333 sleep state data.

### 2.2. Feature Extraction Process

In this experiment, all features were extracted from the PPG signal, and these features can be divided into, time-domain features, frequency-domain features, and nonlinear domain features.

#### 2.2.1. Time-Domain Feature Extraction of PPG Signal

The time-domain characteristics of the PPG signal can intuitively reflect the changes of sleep stage with time. The time-domain features extracted in this paper are shown in Table 2, where Z stands for PPG data.

#### 2.2.2. Frequency-Domain Features

The frequency-domain characteristics of the heart rate variability signal extracted from the PPG signal clearly reflect the activity of the human autonomic nervous system. The features include the frequency band power of each frequency band of heart rate variability. According to the PPG signal, the R-R intervals can be accurately obtained, thereby reliably obtaining heart rate variability. Heart rate variability is usually obtained by ECG signals. In this study, there is a strong correlation between the heart rate variability signals obtained by PPG signals and the heart rate variability signals extracted by ECG signals using PSG datasets, which has also been proven by some scholars [22]. The frequency band of heart rate variability can be divided into low frequency (LF.0.04–0.15) and high frequency, (HF.0.15–0.4), where LF can be further divided into true low frequency (TLF.0.04–0.1) and medium frequency (MF.0.1–0.15). The power of LF and HF bands is related to the regulation of the sympathetic nervous system (SNS) and parasympathetic nervous system (PNS), respectively.

#### 2.2.3. Nonlinear Features

The information extracted by frequency-domain features and time-domain features was still limited, so this experiment introduced many advanced nonlinear feature extraction methods to further extract PPG signal features. Since the PPG signal sampling time at each sleep stage is only 30 s, this paper adopted a nonlinear feature extraction method suitable for short-term PPG signals. These methods included, approximate entropy (ApEn), sample entropy (SampEn), fuzzy entropy permutation entropy, and recurrence Plot.

In order to solve the difficulty of solving entropy in chaos, Pincus proposed the concept of ApEn analysis: an indicator used to measure the complexity of time series from nonlinear time series [23,24]. The theoretical implementation of the ApEn algorithm is shown below:

Perform m-dimensional spatial reconstruction on an N-dimensional time series $\left[u\left(1\right),u\left(2\right),\ldots ,u\left(N\right)\right]$ obtained by sampling at equal time intervals. The reconstructed *i-*th vector is expressed as Equation (1):

(1) $$X\left(i\right)=\left[u\left(i\right),u\left(i+1\right),\ldots ,u\left(i+m-1\right)\right]$$

For 1≤i≤N−m+1, calculate the number of vectors that satisfy the following Formula (2). Given the threshold *r*, usually *r*  =  0.1~0.25 SD (SD is the standard deviation of the sequence $X\left(i\right)$:

(2) $$C_{i}^{m}\left(r\right)=\left(number\;of\;x\left(j\right)\;such\;that\;d\left[X\left(i\right),X\left(j\right)\leq r\right]\right)/\left(N-M+1\right)$$

where $d\left[X\left(i\right),X\left(j\right)\right]$ represents the maximum distance between $X\left(i\right)$ and $X\left(j\right)$, *m* is the pre-selected mode dimension. ApEn is calculated as:

(3) $$\Phi^{m}\left(r\right)=\frac{1}{N-m+1}\overset{N-m+1\sum C_{i}^{m}}{\underset{i=1}{\sum }}ln$$

(4) $$ApEn=\Phi^{m}\left(r\right)-\Phi^{m+1}\left(r\right)$$

During the calculation of *ApEn*, the m is set as 2 and the *r* is 0.15 SD.

SampEn is also a method to describe the complexity of time series, which is improved based on the *ApEn* method [25]. It has applications in assessing the complexity of physiological time series and diagnosing pathological state. The SampEn algorithm steps for an original time series $\left[u\left(1\right),u\left(2\right),\ldots ,u\left(N\right)\right]$ are as follows:

Firstly, using the original time series construct a set of m dimensional vectors, where $X\left(i\right)=\left[u\left(i\right),u\left(i+1\right),\ldots ,u\left(i+m-1\right)\right]$. For 1≤i≤N−m+1, calculate the number of vectors that satisfy the following formula [26,27,28]. Define the function:

(5) $$B_{i}^{m}\left(r\right)=\frac{\mathrm{number}\;\mathrm{of}\;X\left(j\right)\;\mathrm{such}\;\mathrm{that}\;d\left[X\left(i\right),X\left(j\right)\right]\leq r}{N-m+1},j\neq i$$

(6) $$B^{m}\left(r\right)={\left(N-m+1\right)}^{-1}\overset{N-m+1}{\underset{i=1}{\sum }}B_{i}^{m}\left(r\right)$$

Then, define another function, let *k* = *m* + 1:

(7) $$A_{i}^{k}\left(r\right)=\frac{\;\mathrm{number}\;\mathrm{of}\;X\left(j\right)\;\mathrm{such}\;\mathrm{that}\;d\left[X\left(i\right),X\left(j\right)\right]\leq r}{N-m+1},j\neq i$$

(8) $$A^{k}\left(r\right)={\left(N-k+1\right)}^{-1}\overset{N-k+1}{\underset{i=1}{\sum }}B_{i}^{k}\left(r\right)$$

For a finite dataset, the SampEn is estimated as follows:

(9) $$\mathrm{SampEn}\;=-ln\left[A^{k}\left(r\right)/B^{m}\left(r\right)\right]$$

Fuzzy entropy is similar to the physical meaning of *ApEn* and SampEn. It measures the magnitude of the probability that the new model produces. The larger the measure, the greater the probability that the new pattern will produce, meaning that the sequence complexity is greater. The fuzzy entropy algorithm steps for an original time series $\left[u\left(1\right),u\left(2\right),\ldots ,u\left(N\right)\right]$ are as follows [29,30]:

Firstly, using the original time series construct a set of *m* dimensional vectors, where $X\left(i\right)=\left[u\left(i\right),u\left(i+1\right),\ldots ,u\left(i+m-1\right)\right]-\frac{1}{m}\sum_{j=0}^{m-1}u\left(i+j\right),j=1,2,\ldots ,N-m+1$. Then, add fuzzy membership function:

(10) $$A\left(x\right)=\left\{\begin{array}{cc} 1, & x=0\\ exp\left[-ln(2){\left(\frac{x}{r}\right)}^{2}\right], & x>0 \end{array}\right.$$

(11) $$A_{ij}^{m}=exp\left[-ln(2)*{\left(\frac{d_{ij}^{m}}{r}\right)}^{2}\right],j=1,2,\ldots ,N-m+1,\;\mathrm{and}\;j\neq i$$

(12) $$C_{i}^{m}\left(r\right)=\frac{1}{N-m}\overset{N-m+1}{\underset{j=1,j\neq i}{\sum }}A_{ij}^{m}$$

(13) $$\Phi^{m}\left(r\right)=\frac{1}{N-m+1}\overset{N-m+1}{\underset{i=1}{\sum }}C_{i}^{m}\left(r\right)$$

Therefore, the fuzzy entropy of the original time series is as follows:

(14) $$\mathrm{FuzzyEn}\;\left(m,r\right)=\underset{N\to \infty }{lim}\left[\ln\Phi^{m}\left(r\right)-\ln\Phi^{m+1}\left(r\right)\right]$$

For a finite dataset, the fuzzy entropy is estimated as follows:

(15) $$\mathrm{FuzzyEn}\;\left(m,r,N\right)=\ln\Phi^{m}\left(r\right)-\ln\Phi^{m+1}\left(r\right)$$

where $d_{ij}^{m}=d\left[X\left(i\right),X\left(j\right)\right]$ represents the maximum absolute distance between the window vectors $X\left(i\right)$ and $X\left(j\right)$, r is the similar tolerance limit, and m is the preselected modal size. The paper takes m=2 and r=0.15SD.

The permutation entropy is the same as the *ApEn*, SampEn, and fuzzy entropy mentioned above, and is an indicator for measuring the complexity of time series. The difference is that it introduces the idea of permutation when calculating the complexity between reconstructed subsequences [31]. The permutation entropy algorithm steps for an original time series $\left[u\left(1\right),u\left(2\right),\ldots ,u\left(N\right)\right]$ are as follows: First, phase space reconstruction of time series X (phase space size is denoted as m) yields a matrix: rearrange the ascending order of each row of the reconstructed matrix, and if the same two values are encountered, arrange them according to the subscript, thus generating a sequence of symbols. Finally, the number of occurrences of row subscript order is calculated as the row probability, and the entropy of arrangement is the sum of the entropy of all rows in the time series.

The recurrence plot method is an innovative tool for analyzing periodicity and nonlinearity of time series, and it can dig out the internal structure of time series. The recursive graph is used in the experimental data by Eckmann et al. [32,33] and its definition is as follows:

(16) $$R_{i,j}=\Theta \left(\varepsilon_{i}-\|x_{i}-x_{j}\|\right),x_{i}\in R^{m},i,j=1,\ldots ,N$$

Recurrence rate is the density of recursive points in a recursive graph, which is the percentage of recursive points (the proportion of the total number of black points in the recursive graph); determinism is the percentage of recursive points that form a diagonal in the recursive graph (the proportion of black points on the line segment that constitutes the parallel diagonal direction). The measure is defined as follows:

(17) $$DET=\frac{\sum_{l=l_{min}}^{N\sum }l}{\sum_{i,j}^{N}R_{i,j}}$$

#### 2.2.4. Summary of PPG Features

A total of 27 features were explored and are summarized in Table 3. 

### 2.3. Classification Procedures

Following the completion of the above preparation phase, the feature dataset was standardized and subjected to leave-one-out cross-validation. Afterward, the datasets were classified by using the LightGBM and the sleep staging process was performed.

LightGBM is a gradient lifting algorithm based on Gradient Boosting Decison Tree (GBDT) [34]. Its main improvement measures include histogram algorithm and leaf-wise decision tree growth strategy with depth limitation. The decision tree submodel in LightGBM splits the nodes by tiling. Therefore, compared with XGBoost [35], its computational cost is small. We must control the depth of the tree and the minimum data of each leaf node to avoid the fitting phenomenon. The histogram-based decision tree algorithm divides the feature values into multiple kegs and then searches for optimal partitions on those buckets, thereby reducing storage and computational costs. This enhances the robustness to noise while ensuring good evaluation accuracy and training speed. It is proposed to solve the problems encountered by GBDT in massive data so that GBDT can be better applied to reality.

First, given the training set $X={\left\{\left(x_{i,}y_{i}\right)\right\}}_{i=1}^{n}$, the purpose of the LightGBM algorithm is to find a suitable $\overset{¯}{p}\left(x\right)$, as close as possible to $p^{*}(x)$, to minimize the expected value of the specific loss function $L\left(y,p\left(x\right)\right)$, as follows:

(18) $$\overset{¯}{p}\left(x\right)=argmin{\;}_{p}E_{y,X}L\left(y,p\left(x\right)\right)$$

LightGBM integrates a large number of *T*-regression trees $\sum_{t=1}^{T}p_{t}\left(X\right)$, which can be approximated to the final model:

(19) $$p_{T}\left(X\right)=\overset{T}{\underset{t=1}{\sum }}p_{t}\left(X\right)$$

The regression tree should be represented as $W_{q\left(x\right)},q\in \left\{1,2,\ldots ,J\right\}$ where J is the number of leaves, q is the decision rule of the tree, and w is a vector of leaf node sample weights. Hence, LightGBM is trained in the following form:

(20) $$\varphi_{t}=\overset{n}{\underset{i=1}{\sum }}L\left(y_{i},P_{t-1}\left(x_{i}\right)+p_{t}\left(x_{i}\right)\right)$$

The objective function is a fast approximate-place Newton method. For the sake of simplicity, the constant term in (21) is removed and the formula becomes:

(21) $$\varphi_{t}≅\overset{n}{\underset{i=1}{\sum }}\left(L\left(g_{i},p_{t}\left(x_{i}\right)+\frac{1}{2}h_{i}p_{t}^{2}\left(x_{i}\right)\right)\right.$$

where $g_{i}$ represents first-order statistics of loss function, $h_{i}$ represents second-order statistics of loss function, and $I_{j}$ is the sample set of the leaf j, and then (22) can represent the following formula:

(22) $$\varphi_{t}=\overset{j}{\underset{i=1}{\sum }}\left(\left(\underset{i\in I_{j}}{\sum }g_{i}\right)w_{j}+\frac{1}{2}\left(\underset{i\in I_{j}}{\sum }h_{i}+\gamma \right)w_{j}^{2}\right)$$

For the structure *q*(*x*) of the tree, the optimal leaf weight fraction of each leaf node $w_{j}^{*}$ and the extreme value of $\varphi_{T}^{*}$ can be solved as:

(23) $$w_{j}^{*}=-\frac{\sum_{i\in I_{j}}g_{i}}{\sum_{i\in I_{j}}h_{i}+\gamma }$$

(24) $$\varphi_{T}^{*}=-\frac{1}{2}\overset{J}{\underset{j=1}{\sum }}\frac{{\left(\sum_{i\in I_{j}}g_{i}\right)}^{2}}{\sum_{i\in I_{j}}h_{i}+\gamma }$$

$\varphi_{T}^{*}$ is an important scoring function of the tree structure *q*, then the objective function can be expressed as:

(25) $$G=\frac{1}{2}\left(\frac{{\left(\sum_{i\in I_{L}}g_{i}\right)}^{2}}{\sum_{i\in I_{L}}h_{i}+\gamma }+\frac{{\left(\sum_{i\in I_{R}}g_{i}\right)}^{2}}{\sum_{i\in I_{R}}h_{I}+\gamma }+\frac{{\left(\sum_{i\in I}g_{i}\right)}^{2}}{\sum_{i\in I}h_{i}+\gamma }\right)$$

where $I_{L}$ and $I_{R}$ are the left and right branches of the sample set, and LightGBM will allow the tree to grow vertically, which will be more efficient when processing large amounts of data.

### 2.4. Decision Mechanism

In the field of machine learning, accuracy is the most basic statistical classification evaluation indicator, but it cannot fully demonstrate model performance. In order to visualize the performance of the sleep staging algorithm objectively and comprehensively, the confusion matrix, recall rate and F1 score were used as evaluation criteria. In addition to statistical criteria, Cohen’s kappa coefficient is used to represent the correlation of sleep staging results [36].

## 3. Results and Discussion

This study used PPG data extracted from the CAP sleep database, which was derived from 27 subjects, 4 of whom were healthy, 5 had insomnia, 10 had nocturnal frontal lobe epilepsy, and 8 had REM behavior disorder. First of all, 27,333 periods were obtained after data preprocessing such as data cleaning, data filtering, and data denoising. In order to make the final result more authoritative, this study performed a balanced processing of various types of data according to multimodal staging criteria.

After preprocessing, this study extracted features from the time domain, frequency domain, and nonlinear domain and obtained 21 features in total. After effective features were determined, the feature dataset were normalized, and the dataset was divided using the 10-fold cross-validation method. Finally, the training set was used to train the LightGBM classifier for sleep staging, the validation set was used to adjust the model, and the test set was used to evaluate the model according to the above evaluation criteria.

In this experiment, four evaluation indexes including accuracy, recall rate, F1 score, and Cohen’s kappa statistic k were used to evaluate the performance of the model. The results of multi-class sleep staging in the test dataset are shown in Table 4. Among them, the accuracy rate of the 3-class was higher than 86%, and Cohen’s kappa statistic k was also higher than 0.79, which was highly similar to the expert scoring results. The sleep staging results of the 4-class and the 5-class were slightly inferior to the sleep staging results of the 3-class, but the accuracy rate was also higher than 72% and the Cohen’s kappa statistic k coefficient was also higher than 0.6, which basically meets the accuracy requirements of most sleep staging scenes. From the experimental results, the PMSS method still lacks the ability of multi-class sleep classification compared with the ability of multi-class sleep staging using EEG signals. However, compared with other single-channel physiological signals for sleep classification, such as ECG and respiratory signals, the accuracy was significantly improved.

**Figure 3:**
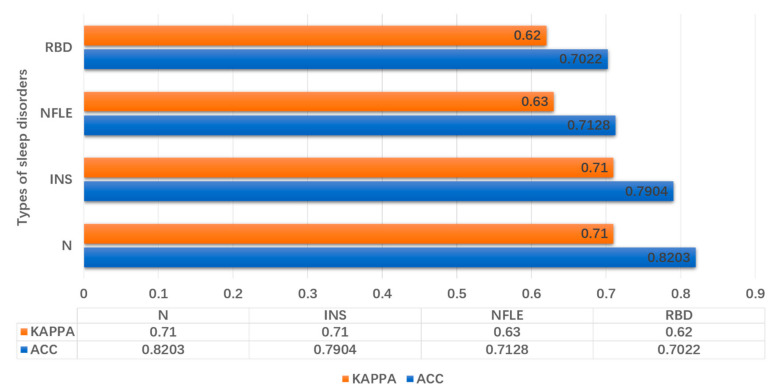
The ability of the model to classify data of different diseases.

The divided test dataset contained a mixed dataset of some data from healthy subjects, insomnia subjects, nocturnal frontal lobe epilepsy subjects, and REM behavior disorder subjects. In order to verify the classifying ability of the experimental model for subjects with sleep disorders, this article used four different health conditions of the subject data to perform 4-class sleep staging. The results of sleep staging are shown in Figure 3. Among them, the sleep staging ability is the best for healthy people, with an accuracy rate of more than 80%. The sleep staging ability of the subjects with the disease was decreased, but the consistency is more than 0.60. It can be concluded that the model is still suitable for sleep staging of subjects with sleep disorders.

Compared with articles using PPG signals for sleep classification in recent years, this experiment only uses single-channel PPG signals as classification data and does not require the assistance of other signals, which greatly reduces the impact of the experiment on the subjects’ natural sleep. Judging from the classification results, the accuracy and Cohen’s kappa statistic k of sleep staging in 3-class, 4-class, and 5-class in this experiment are higher than those of the current study of sleep classification using PPG signals. Not only that, the experimental sleep classification model has a strong generalization ability and can meet the sleep staging needs of patients with sleep diseases. Nevertheless, this experiment still encountered some problems. The collection of PPG signals is based on the principle of light reflection. Therefore, when this method is used at a high light intensity, large errors will occur, which will become a problem to be solved in the next step.

## 4. Conclusions

PMSS method was proposed with only PPG signal used to stage the sleeping status. PMSS method can achieve 3-class, 4-class, and 5-class sleep staging, and the results of multi-class sleep staging are highly consistent with the results of manual sleep staging conducted by several experts based on PSG signals. The reason is that because PPG signals can extract HRV signals and SpO2 signals, they have all the information of these signals. At the same time, these signals have been recognized by many scholars as suitable for sleep staging. It is well understood that PPG signals can be obtained in sleep staging experiments. In addition, this method can also achieve a consistent result on PPG data of subjects with sleep disorders. Therefore, this study believes that the PMSS method has generalization ability and can be applied to home sleep monitoring for patients with sleep disorders and healthy subjects, greatly reducing human resource consumption and reducing the impact on the subject during sleep monitoring. In the next step of this study, considering the accuracy of this method, we will try to apply this method to the diagnosis of sleep disorders.

## Figures and Tables

**Table 1:** Subject’s personal information.

Pathology	Male	Female	Age	Identification Number
No pathology	1	3	28–35	n2, n3, n5, n11
Nocturnal frontal lobe Epilepsy	5	5	14–41	nfle1–nfle10
Insomnia	1	4	47–64	ins2, ins5–ins8
REM behavior disorder	7	1	70–82	ins2–ins9

**Table 2:** The symbols and meanings of time-domain features.

Name	Meaning	Formula
Med_PPG	median	$Median\;(Z_{i})$
Max_PPG	maximum value	$Max\;(Z_{i})$
Min_PPG	minimum value	$Min\;(Z_{i})$
Dif_PPG	difference between the maximum and the minimum	$Max\;(Z_{i})-Min\;(Z_{i})$
Var_PPG	variance	$\frac{1}{n}\overset{n-1}{\underset{j=0}{\sum }}\;(Z_{ij}-\bar{Z_{i}}{)}^{2}$
Ske_PPG	Coefficient of skewness	$E[\left(\frac{Z_{ij}-m}{r}{)}^{3}\right]$
Kur_PPG	Coefficient of kurtosis	$E[\left(\frac{Z_{ij}-m}{r}{)}^{4}\right]$
Mean_PPG	mean value	$\frac{1}{n}\overset{n-1}{\underset{j=0}{\sum }}Z_{ij}$
En1st_PPG	Comentropy of first order difference	$Comentropy\left(1st\right)$
En2nd_PPG	Comentropy of Second order difference	$Comentropy\left(2nd\right)$
En1st_2nd_PPG	Comentropy of first-order difference divided by entropy of second-order difference	$\frac{Comentropy\left(1st\right)}{Comentropy\left(2nd\right)}$

**Table 3:** Summary of photoplethysmography (PPG) features.

Category	Name	Number
Time domain	Mad_PPG, Max_PPG, Min_PPG, Dif_PPG, Var_PPG, Ske_PPG, Kur_PPG, Mean_PPG, En1st_PPG, En2st_ PPG, En1st_2st_PPG	11
Frequency domain	LF_power, TLF_power, HF_power, MF_power	4
Nonlinear analysis	ApEn, SampEn, FuzzyEn, PerEn, DET, Recurrence Rate	6
In total		21

**Table 4:** Confounding matrix and evaluation index of multiple sleep stages.

3-Class											
Predicted result by the proposed method											
Clinical analysis result		W	NREM			REM			Precision	Recall	F1-score
	W	342	36			14			0.82	0.87	0.84
	NREM	51	353			14			0.85	0.84	0.85
	REM	26	24			340			0.92	0.87	0.90
	Accuracy: 0.8625							Cohen’s kappa statistic k: 0.79			
**4-Class**											
Predicted result by the proposed method											
Clinical analysis result		W	LS		SWS		REM		Precision	Recall	F1-score
	W	360	25		20		6		0.81	0.88	0.84
	LS	36	276		40		34		0.70	0.72	0.71
	SWS	25	48		308		40		0.78	0.73	0.75
	REM	21	43		29		289		0.86	0.76	0.77
	Accuracy: 0.7706							Cohen’s kappa statistic k: 0.69			
**5-Class**											
Predicted result by the proposed method											
Clinical analysis result		W	N1	N2		N3	REM		Precision	Recall	F1-score
	W	193	1	11		14	11		0.78	0.84	0.81
	N1	11	38	7		16	22		0.75	0.40	0.52
	N2	13	4	156		21	10		0.68	0.76	0.72
	N3	12	1	39		156	13		0.68	0.71	0.69
	REM	18	7	17		23	160		0.74	0.71	0.73
	Accuracy: 0.7217							Cohen’s kappa statistic k: 0.64			

## Data Availability

Publicly available datasets were analyzed in this study. This data can be found here: https://www.physionet.org/content/capslpdb/1.0.0/.
